# Guipi decoction for coronary heart disease: A protocol for a systematic review and meta-analysis

**DOI:** 10.1097/MD.0000000000021589

**Published:** 2020-08-07

**Authors:** Huanjia Gao, Shiyu Liu, Hairong Cai, Dongjie Chen, Xue Fu, Shuai Zhao, Bojun Chen

**Affiliations:** a The Second Clinical Medical School, Guangzhou University of Chinese Medicine; b Department of Emergency, the Second Affiliated Hospital of Guangzhou University of Traditional Chinese Medicine; c The First Clinical Medical School, Guangzhou University of Chinese Medicine; d Department of Critical Care Medicine, Beijing University of Chinese Medicine Shenzhen Hospital, Shenzhen, Guangdong, Province, China.

**Keywords:** Guipi decoction, coronary heart disease, protocol, systematic review

## Abstract

**Background:**

Coronary heart disease (CHD) is the leading cause of morbidity and mortality worldwide. Guipi Decoction (GPD) is a classical traditional Chinese medication used to treat CHD. However, systematic review and meta-analysis regarding its efficacy and safety has not been systematically evaluated. The objective of this protocol is to determine the efficacy and safety of GPD in the treatment of CHD.

**Methods:**

Randomized controlled trials evaluating the effectiveness and safety of GPD in the treatment of CHD will be retrieved from 8 electronic databases, including PubMed, EMBASE, Cochrane Library, Web of science, China National Knowledge Infrastructure Database, VIP Database, Wanfang Database and China Biology Medicine Database. Study selection, data collection, risk of bias assessment, and evaluation of the quality of evidence will be performed in order. Data will be analyzed by RevMan V.5.3.5 software.

**Results:**

This study will evaluate the efficacy and safety of GPD in the treatment of CHD.

**Conclusion:**

This systematic review will provide evidence for determining whether or not GPD is an effective and safe intervention for CHD.

**PROSPERO registration number:**

PROSPERO CRD 42020156420.

## Abstract 2

Supplemental Digital Content is available in the text

## 1 Introduction

Coronary Heart Disease (CHD) a serious cardiovascular disease of myocardial ischemia or necrosis caused by stenosis or occlusion by coronary atherosclerosis.^[1,2]^ In China, the prevalence and mortality of CHD have increased year by year,^[3–6]^ with the aging of the global population, the incidence of CHD rises year by year. CHD causing serious economic and social burden,^[7,8]^ has become a major public health problem in the world.^[9–11]^ CHD is still the number one cause of death worldwide, exceeding the sum of all tumor causes.^[12]^ At present, the mortality rate of CHD also accounts for the first cause of total death in China. The total cost of hospitalization for AMI was as high as 13.375 billion Yuan.^[13]^

Currently, the main treatment for CHD mainly includes drug therapy, percutaneous coronary intervention (PCI), coronary artery bypass grafting (CABG).^[14–17]^ However, it could result in certain side effects and poor compliance. Traditional Chinese medicine (TCM) is an important part of complementary and alternative medicine (CAM), which has been widely accepted in China and applied in practice.^[18]^ GPD is composed of 12 kinds of TCM (Atractylodes macrocephala, Panax ginseng, Astragali Radix, Angelica Licorice, Glycyrrhizae Radix, Poria, Polygalae Radix, Ziziphi Spinosae Semen, Radix Aucklandiae, Dimocarpus longan Lour). GPD has been often used in the treatment of CHD in clinical practice in China with uncertain effects.^[19,20]^ However, to our knowledge, there is no systematic review of its efficacy and safety in the treatment of CHD. Therefore, we propose the current protocol to evaluate the effectiveness and safety of GPD on CHD, providing a reference for clinical use.

## 2 Methods

### 2.1 Inclusion criteria for study selection

#### 2.1.1 Types of studies

All relevant randomized controlled trials (RCTs) regarding GPD for the treatment of CHD will be involved without limitations on language, publication or blinding.

#### 2.1.2 Types of patients

We will include patients with CHD, including stable angina pectoris (SAP), unstable angina (UA), non-st-segment elevation myocardial infarction (NSTEMI), and st-elevation myocardial infarction (STEMI). There is no limit to sex, ethnicity, education, economic status, and disease severity.

#### 2.1.3 Types of interventions

The control group was treated with conventional treatment, including clopidogrel, aspirin, angiotensin-converting enzyme inhibitor (ACEI), low molecular weight heparin, beta blockers, statins, nitrates, and combined treatment of GPD and conventional treatment was used in the experimental group.

We will include various dosage forms of GPD, including tablets, capsules, pills, powders and extracts. We will exclude RCTs in which GPD is combined with other Chinese medicine methods, such as acupuncture and moxibustion. There is no limit to dose and route of administration.

#### 2.1.4 Types of outcome measures

##### 2.1.4.1 Primary outcomes

The primary outcomes will be major adverse cardiovascular events (MACE), including nonfatal myocardial infarction, nonfatal cardiogenic shock, coronary revascularization, coronary heart disease death, and severe arrhythmias.

##### 2.1.4.2 Secondary outcomes

Total effective rate of electrocardiogram, high sensitive C reaction protein (hs-CRP), Interleukin-6 (IL-6), matrix metalloproteinases-9 (MMP-9), creatine kinase isoenzyme (CK-MB), myocardial troponin I(cTnI), B-type natriuretic peptide (BNP), blood lipids, hematocrit, fibrinogen, angina pectoris attacks and intervals, left ventricular diastolic End-stage diameter, left ventricular end-systolic diameter, left ventricular ejective fraction (LVEF), cardiac output, cardiac index, cardiac per volume, and adverse drug reactions.

### 2.2 Search methods for the identification of studies

We will perform a comprehensive literature search of relevant databases including Cochrane Library, PubMed, EMBASE, WOS, CNKI, WangFang, CBM, and VIP from their inception to June 2020. English search terms include: Guipi decoction, coronary heart disease and RCTs. The strategy for searching the PubMed will be shown as an example in Appendix A (Supplemental Appendix A), and modified by using other databases.

#### 2.2.1 Searching other resources

Google Scholar and Baidu Academic will be involved to search relevant literature. We will also manually retrieve relevant literature from Clinical Trials Register. In addition, reference lists of eligible studies will be performed manually so as to avoid missing vital information.

### 2.3 Data collection and analysis

#### 2.3.1 Selection of studies

Selection of studies will be performed independently by 2 researchers. First, the obvious disqualified literatures will be excluded by screening the titles and abstracts. Secondly, they will determine whether the references up to the

**Figure 1:**
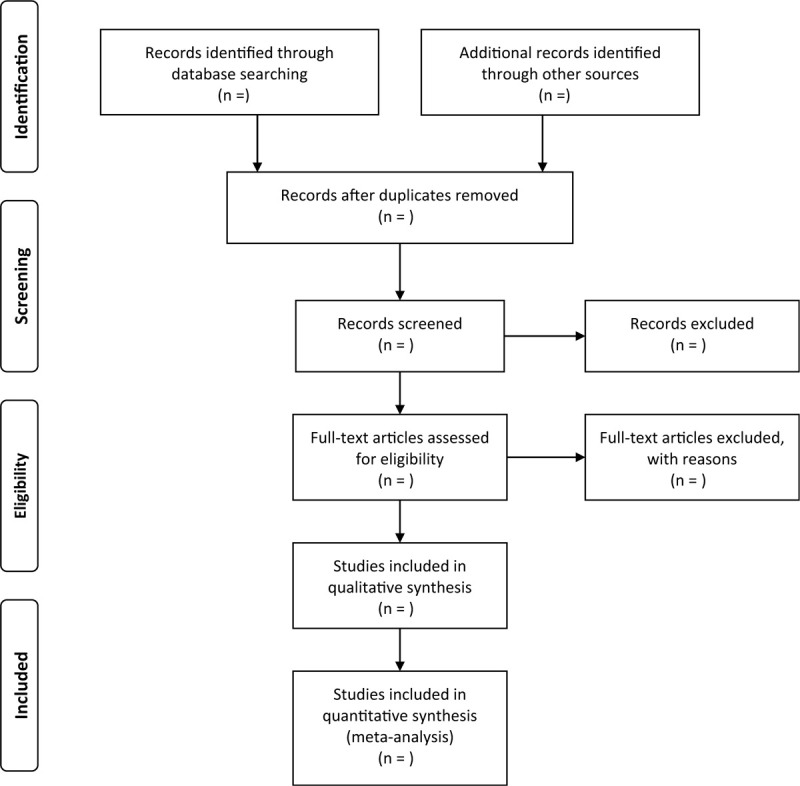
Preferred reporting items for systematic review and meta-analysis (PRISMA) flow chart.

Standards or not by reading through the text. Any disagreements will be resolved by discussion or by consulting a third investigator if needed. The process of studies selection and meta-analysis is presented in a in an adapted PRISMA flow diagram (Fig. 1).

#### 2.3.2 Data extraction and management

Data extraction and management will be performed by 2 independently researchers. A standard data extraction form, including author, year of publication, ethnicity, diagnostic criteria of CHD, number of cases and controls, interventions, measurement indicators, results, and adverse events, will be created before data extraction. Any disagreement will be settled after consulting a third researcher or by discussion.

#### 2.3.3 Assessment of risk of bias in included studies

The “risk of bias” tool recommended by the Cochrane Handbook V.5.3 will be used to assess the risk of bias by 2 researchers independently. The main items include random sequence generation, allocation concealment, blinding, incomplete outcome data, selective reporting, and other biases. The results of the assessment will be divided into 3 levels, such as “low risk,” “high risk,” or “uncertain”. A consensus will be reached through discussion and consultation with a third reviewer if there are any inconsistencies in the assessment.

#### 2.3.4 Measures of treatment effect

Analysis will be based on available data of included studies, the relative data (RR) or odds ratio (OR) with 95% confidence interval (CI) will be used to analyze dichotomous data. While the standardized mean difference (SMD) or weighted mean difference (WMD) with 95% CI will be used for continuous data.

#### 2.3.5 Dealing with missing data

We will contact the first or corresponding author to get missing information from their trials and use the available data for data synthesis. If the necessary data are unobtainable, the impact of missing data will be discussed.

#### 2.3.6 Assessment of heterogeneity

*I*^2^ statistic and *x*^2^ test will be used to evaluate the heterogeneity. The heterogeneity will be divided into 3 levels: *I*^2^ < 25% will be considered as no statistical heterogeneity;25% < *I*^2^ < 50% means that there is moderate heterogeneity; while *I*^2^ ≥ 50% will be taken as large heterogeneity, hence, subgroup or sensitivity analysis will be conducted.

#### 2.3.7 Assessment of reporting bias

Funnel plot will be performed to evaluate the publication bias when there are 10 or more publications included in this study.

#### 2.3.8 Data synthesis

RevMan software (Version 5.3, Copenhagen: The Nordic Cochrane Center, 2014) will be used to perform data synthesis. The fixed effects model will be performed for meta-analysis when there is small homogeneity (*I*^2^ < 50%). If not, the random effects model will be conducted. Subgroup analysis, sensitivity analysis or descriptive analysis will be performed if significant heterogeneity between studies is found.

#### 2.3.9 Subgroup analysis

Subgroup analyses will be conducted based on different factors, including interventions, participants, dose of medication, kinds of GPD, and gender.

#### 2.3.10 Sensitivity analysis

Sensitivity analysis will be conducted if there are sufficient data available.

#### 2.3.11 Grading the quality of evidence

The quality of evidence will be evaluated using the grading of recommendations assessment, development and evaluation (Version 3.6, The GRADE Working Group, 2010). The quality of evidence was divided into 4 levels: high, medium, low, and extremely low.

## 3 Discussion

With the aging of the population and the acceleration of urbanization, CHD has become a serious public health problem, which seriously affects human life and health. Drugs, PCI and CABG are the most important treatments for treating CHD. However, there are certain side effects about these treatments. GPD may be a useful treatment for primary insomnia, and it is unlikely to produce severe side effects. As far as we know, it is unclear whether GPD is effective and safe intervention for CHD. Therefore, we aim at providing evidence to clinicians so that more and more patients with CHD may also benefit from alternative interventions. However, there are some certain potential limitations in this systematic review. First, the language is limited to Chinese or English, which may result in selection bias. Second, different dosage of herbs, the age of the patient, and the severity of CHD may present a heterogeneity risk. Finally, small samples of RCTs may lead to high risks of bias.

## Author contributions

**Conceptualization:** Huanjia Gao.

**Data curation:** Shuai Zhao.

**Funding acquisition:** Bojun Chen.

**Investigation:** Xue Fu.

**Methodology:** Dongjie Chen.

**Project administration:** Bojun Chen.

**Software:** Shiyu Liu.

**Supervision:** Bojun Chen.

**Validation:** Hairong Cai.

**Writing – original draft:** Huanjia Gao.

**Writing – review & editing:** Huanjia Gao, Shiyu Liu.

## Supplementary Material

Supplemental Digital Content
