# Supplementary material for: Guipi decoction for coronary heart disease: A protocol for a systematic review and meta-analysis
Source: Medicine (Baltimore). 2020 Aug 7;99(32):e21589. doi: 10.1097/MD.0000000000021589 (PMC7593082; doi:10.1097/MD.0000000000021589)
Supplement: Supplemental Digital Content [file medi-99-e21589-s001.docx]

**Appendix A.**

***Search strategy used in PubMed database***

#1 Coronary heart disease OR Coronary Diseases OR Disease, Coronary

OR Disease, Coronary Heart OR Heart Disease, Coronary

#2 Guipi Decoction OR gui pi decoction OR gui pi tang OR gui pi yin

#3 Randomized controlled trial OR clinical study OR Clin-ical Trial OR Controlled study OR Controlled Trial OR Random*Control* study OR random* Control* Trial

#1 AND #2 AND #3
